# H_2_O_2_/Glucose Sensor Based on a Pyrroloquinoline Skeleton-Containing Molecule Modified Gold Cavity Array Electrode

**DOI:** 10.3390/nano12101770

**Published:** 2022-05-23

**Authors:** Kaiyue Wang, Xuefang Gu, Qun Zhao, Xinyi Shao, Yaqi Xiao, Chongyu Zhong, Shu Tian, Bing Yang

**Affiliations:** School of Chemistry and Chemical Engineering, Nantong University, Nantong 226019, China; 1608022036@stmail.ntu.edu.cn (K.W.); qunzhao@ntu.edu.cn (Q.Z.); 1908110265@stmail.ntu.edu.cn (X.S.); 1908110186@stmail.ntu.edu.cn (Y.X.); 1908110353@stmail.ntu.edu.cn (C.Z.)

**Keywords:** gold cavity array, electrochemical sensor, glucose, hydrogen peroxide, pyrroloquinoline skeleton

## Abstract

H_2_O_2_-related metabolites are essential indicators in clinical diagnosis because the accumulation of such reactive oxygen species could cause the risk of cardiovascular disease. Herein, we reported an electrochemical sensor to determine H_2_O_2_ and glucose. The pyrroloquinoline skeleton containing molecules (PQT) were used as the electrocatalyst and the gold cavity array (GCA) electrodes as the supporting electrode. The GCA electrode was fabricated by electrodeposition using high-ordered two-dimensional polystyrene spheres as the template. The strong absorbability of iodide ions (I^−^) displaced adventitious materials from the metal surface and the I^−^ monolayer was subsequently removed by electrochemical oxidation to get a clean electrode surface. PQT molecules were firmly immobilized on the GCA electrode and performed an excellent electrocatalytic effect on H_2_O_2_/glucose detection, manifested by a small overpotential and a significantly increased reduction current. A good linear correlation was observed over a wide range of 0.2 μmol/L–1.0 mmol/L with the limit of detection of 0.05 μmol/L. Moreover, the sensor can realize sensitive, accurate, and the highly selective detection of actual samples, proving its application prospect in clinical diagnosis.

## 1. Introduction

Enzymes (for example, NADH dehydrogenase and cytochrome c oxidase) in the human electron transfer chain have a low probability of transferring electrons to oxygen to generate various reactive oxygen species (ROS) [1,2,3]. Appropriate concentrations of ROS can correctly activate cellular antioxidant defense mechanisms and ROS can participate in immune and signal transduction processes in vivo [4,5], while the accumulation of ROS can cause the risk of cardiovascular disease and even cancer [6]. Several different approaches have been developed for the detection of ROS in vivo or in vitro [7,8], mainly electrochemical and optical methods, including fluorescence [9,10], surface plasmon resonance [11], and Raman spectroscopy [12,13]. However, each method has disadvantages, such as excessive background signals, tedious sample processing, high running cost, etc., which restrict their widespread application. Therefore, developing a convenient, sensitive, and accurate method for reactive oxygen species detection is of great importance for disease diagnosis and therapeutic evaluation. Electrochemical methods have attracted a lot of attention among these analytical methods due to their outstanding advantages, such as easy operation, on-site detection, high sensitivity, and low cost [14,15].

Hydrogen peroxide (H_2_O_2_), a byproduct of cellular metabolism and enzymatic reactions in living organisms, and the most typical reactive oxygen species, is an important indicator in biochemical analysis and clinical testing [16]. H_2_O_2_ can be irreversibly reduced on a gold electrode surface, but the relatively large overpotential makes it necessary to catalyze the reduction by adding peroxidase to achieve rapid electron transfer. However, enzymes are susceptible to environmental conditions and are readily inactivated, and enzyme-catalyzed reactions need specific requirements, significantly affecting the detection efficiency. Significant progress has been made in electrochemical research based on metal nanomaterials and small molecule catalysis. These electrocatalytic materials, so-called nanoenzymes, and these nanomaterials, including noble metals, transition metal oxides, and carbon-based materials, and members of the nanoenzyme family, can essentially replace expensive and specific enzymes conducive to the accurate detection of H_2_O_2_ [17,18,19,20,21]. The introduction of noble metals can enhance the conductivity and surface area of the electrode and can also regulate the electronic state of the active center of organic matter [22]. 

Noble metal nanoparticles are one of the commonly used nanoenzymes. They can be prepared by reducing their metal cations in solution, and the morphology of the nanoparticles can be changed by using different reaction conditions, such as changing reducing agents and adding surfactants. However, such metal sols suffer from surface contamination, susceptibility to aggregation, and poor batch-to-batch reproducibility. The detection ability and data reproducibility of the modified electrodes based on these nanoparticles are often unsatisfactory. Array electrodes are a combination of multiple microelectrodes, which have the advantages of a high mass transfer rate, low electric double layer charging current, and a high signal-to-noise ratio, making them an ideal electrode material. A high-density catalyst adsorbed on the rough surface could enhance the sensitivity and selectivity of the sensors [23,24]. 

Many general approaches have been employed to prepare array structures, including template methods [25], self-assembly techniques [26], and e-beam lithography [27]. The template method takes templates as the main configuration to control, affect, and modify the morphology and determine the properties. Since its first appearance, many research groups have systematically studied this powerful method and completed the construction and modification process [28,29]. For example, Baumberg and Bartlett pioneered the fabrication of two-dimensional sphere segment (SSV) metallic arrays using a template and electrodeposition, and intensely studied their applications in electrochemistry, optical waveguides, and surface-enhanced Raman scattering [30,31,32]. Zhao and colleagues [33] constructed a Ta/Ni bimetallic bowl-shaped array electrode for the amperometric detection of uric acid. Nickel nanoparticles were electrodeposited into the gap of the polystyrene (PS) sphere template to get an enlarged surface area of the electrode, providing good support for the Ta nanoparticles and facilitating the electron transfer in the oxidation of uric acid. Our group has also used a similar method to prepare silica-isolated gold cavity array electrodes to construct an H_2_O_2_ biosensor and immunoassays of biomarker proteins [34,35,36]. 

Glucose, as a polyhydroxy aldehyde monosaccharide, is the main component of carbohydrates in the human body [37]. Glucose can effectively regulate the water–electrolyte and acid–base balance in the body and is one of the main sources of calories for the body [38]. However, a high glucose level in human blood may cause diseases, such as cardiovascular and kidney diseases. It may also lead to severe complications and even death. During metabolism, glucose can be oxidized to gluconic acid by O_2_ under the catalysis of glucose oxidase, while, at the same time, O_2_ is reduced to H_2_O_2_. Thus, glucose levels were determined indirectly by monitoring the amount of H_2_O_2_ during the reaction, because the stoichiometric ratio between them is 1:1. This work proposed a nonenzymic H_2_O_2_ sensor based on a pyrroloquinoline skeleton-containing molecules modified gold cavity array (PQT@GCA) electrode for H_2_O_2_ and H_2_O_2_-related metabolites detection. We first rationally designed and synthesized 2-(9-chloro-7-methyl-3*H*-pyrrolo [3,2-*f*]quinolin-1-yl)-2,3-dihydrobenzo [*d*]thiazole (PQT) and achieved the electrochemical determination of H_2_O_2_-related metabolites through the electrocatalytic effect of the PQT molecules on H_2_O_2_ reduction. The gold cavity array (GCA) electrodes were constructed by electrodepositing gold nanoparticles (AuNPs) to the interstice of two-dimensional close-packed PS templates. In the presence of H_2_O_2_, the PQT molecular probe could catalyze the reduction of H_2_O_2_, and thus a sensitive H_2_O_2_/glucose electrochemical sensor was created. The electrochemical behavior of the PQT@GCA electrode was characterized by cyclic voltammetry (CV) and the electrochemical impedance spectrum (EIS). The presented sensor exhibited high sensitivity and selectivity in the range of 0.2 μmol/L–1.0 mmol/L, with a detection limit of 0.05 μmol/L for glucose detection. Finally, the sensor was applied to detect glucose in real serum samples, and the recovery of the spiked sample verified its great promise in a clinical test.

## 2. Materials and Methods

### 2.1. Materials and Reagents

Phosphorus oxychloride (POCl_3_), diphenyl ether, 2-aminobenzenethiol, and 5-Aminoindole were obtained from the Sinopharm Group (Shanghai). The synthesis procedure of the PQT molecule and the product characterization are in the Supporting Information (SI). Chloroauric acid (HAuCl_4_), glucose, and glucose oxidase (GOD) were purchased from Sigma-Aldrich. All the reagents were of analytical reagent grade and used without further purification.

### 2.2. Preparation of Gold Cavity Array (GCA) Electrodes

PS microspheres with a diameter of 1.5 μm dispersed in water/ethanol and self-assembled into a single-layer close-packed template on the water surface (for details, please refer to our earlier literature [36,39,40]). After the suspended PS microspheres settled, the template was transferred to the surface of a gold-coated (magnetron sputtering, 100 nm thickness) ITO slide. Subsequently, the electrode covered with the PS template was then immersed in the gold plating solution. AuNPs were uniformly electrodeposited into the interstice of the template using a multicurrent step method. After that, the PS template was removed to get the GCA electrode (see SI for detailed experimental steps, the composition of gold plating solution, and the electrochemical parameters).

### 2.3. Fabrication of PQT Molecules Modified GCA Electrodes

The obtained GCA electrodes were immersed in 0.1 mol/L KI solution in the dark for 30 min. Iodine ions, therefore, bind firmly to AuNPs and remove the impurities from the electrode surface. After that, the I^−^-modified electrode was scanned by cyclic voltammetry between 0–1 V in 0.1 mol/L NaClO_4_ solution until the oxidation peak at 0.95 V completely disappeared. This was done to remove the adsorbed I^−^ and obtain a clean surface for analysis.

Then, the cleaned GCA electrode was immersed in 2 mmol/L PQT methanol solution for 12 h to immobilize the PQT molecules on the GCA surface. The as-prepared PQT@ GCA electrode was rinsed thoroughly with methanol and blown dry with argon. Before electrochemical detection, we covered the GCA electrode with a preperforated sticker to keep the electrode area constant, leaving only a 5 mm diameter circular hole exposed to the electrolyte.

### 2.4. Glucose Detection

For the electrochemical detection of H_2_O_2_, the PQT@GCA electrode was used as the working electrode, and different concentrations of H_2_O_2_ were added into a 0.2 mol/L NaClO_4_ solution, where cyclic voltammetry (CV) curves were collected.

For the detection of glucose, 1 mL of glucose solution with different concentrations (0.05 μmol/L–2.0 mmol/L) was first reacted with 1 mg/mL glucose oxidase (10 U) for 10 min, the mixture was detected using the same method as the H_2_O_2_ test, and square wave voltammetry (SWV) curves were collected.

## 3. Results and Discussion

### 3.1. Preparation and Characterization of the GCA Electrode

**Figure 1:**
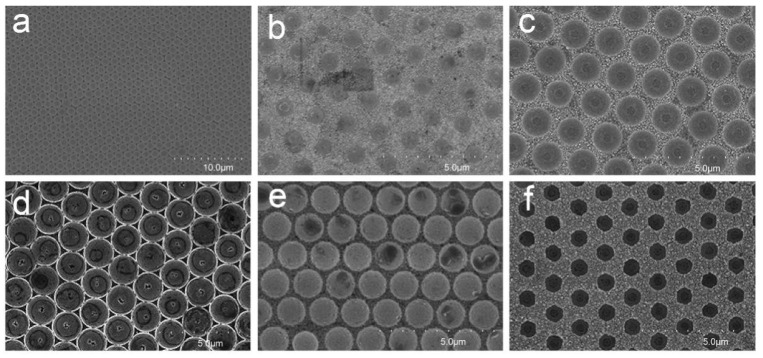
SEM images of (**a**) two-dimensional arrangement PS template with 1.6 µm diameter, (**b**–**f**) GCA with different cavity depths (from (**b**–**f**), 0.3 R, 0.7 R, 1.0 R, 1.2 R, and 1.6 R, respectively) after the template removal.

Monodisperse PS microspheres are used as the two-dimensional ordered template to construct array structures with different functional materials, widely applied in catalysis, sensing, photoelectric components, detection, and analysis. The PS microspheres (1.6 µm in diameter) formed two-dimensional highly ordered template arrays through self-assembly on the gas–liquid interface and then were transferred to the ITO surface. As shown in Figure 1a, the close-packed hexagonal morphology was maintained well after the transference, and no apparent defect was observed. AuNPs were electrodeposited into the interspace between the microspheres. The gold seeds were formed by the first large current pulse and firmly attached to the electrode surface. The subsequent small current pulses made the AuNPs grow evenly around the gold seeds, and the generated GCA was just the casting of the template due to the confinement of the template. The SEM in Figure 1b–f shows the morphology of the GCAs obtained by electrodeposition with an increasing number of small current pulses (500, 1500, 2000, 2500, and 4000 small pulses). The initial stage of the deposition was to fill the gap between the PS spheres and the supporting electrode. The AuNPs were rough at this stage, and the formed cavity depth was shallow and irregular (Figure 1b). The depth of the spherical cavity increased with the extension of the deposition time due to the bottom-up growth manner and the guiding effect of the template (Figure 1c,d). When the number of applied small current pulses was 2000 times, the AuNPs filled the gaps between the templates. They formed a densely arranged spherical cavity array, and the diameter of the opening of the cavity was the same as that of the template PS microspheres (Figure 1d). Further electrodeposition made the array morphology no longer regular, because the subsequently generated AuNPs lost the guiding role of the template (Figure 1e,f). The results show that the morphology of the GCA electrode can be regulated by changing the electrodeposition parameters. This work chooses the GCA with the depth _GCA_ = R_PS_ as the electrode for further PQT modification to obtain a relatively large electrode area and a fast mass transfer rate.

### 3.2. Surface Cleaning of the GCA Electrode

**Figure 2:**
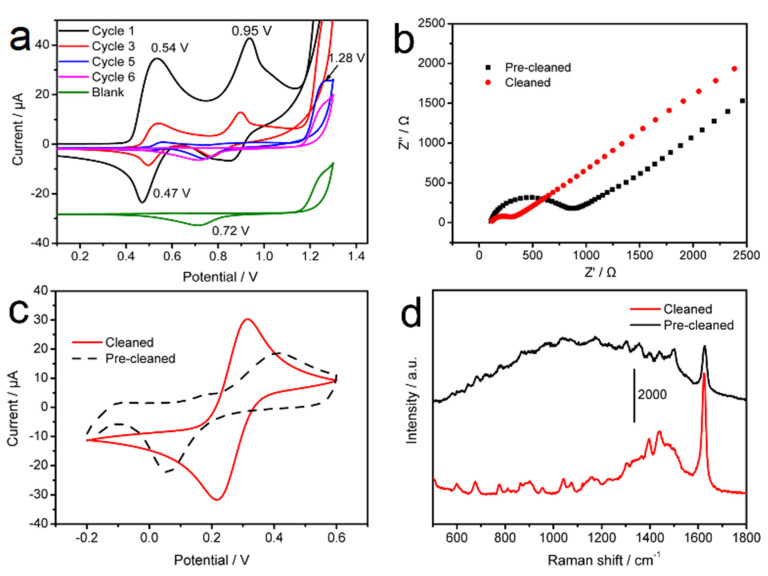
(**a**) CV curves of electrochemical cleaning of GCA electrodes, (**b**) EIS, (**c**) CV curves of [Fe(CN)_6_]^3−/4−^, (**d**) SERS spectra of MB on the GCA substrate before and after the cleaning process.

It should be noted that during the electrode preparation process, some contaminants remain on the electrode surface, including impurities adsorbed from the electrolyte and ambient environment, and the surfactants inherited from the templated PS spheres. The presence of these surface contaminants will inevitably cause background interference, thus limiting the application of this GCA electrode in the electrochemical analysis of the trace samples. Therefore, the GCA electrode needs to undergo a cleaning step to obtain a clean surface for further use. We cannot get clean electrodes by ultrasonic or electrochemical cleaning because severe vibration and long-time electrochemical scanning will peel the thin gold layer from the ITO surface. We thus used the strong adsorbability of I^−^ on Au/Ag to displace adventitious materials from the metal surface, and then removed the I^−^ monolayer by electrochemical oxidation. Figure 2a shows the cyclic voltammograms of a blank GCA electrode and an iodide ions-modified GCA electrode in 0.2 mol/L NaClO_4_. Two strong oxidation peaks were observed at ~0.54 V and ~0.95 V on the I^−^-treated GCA electrode, which could be attributed to the oxidation of I^−^ to I_2_ and I_2_, further being oxidized to IO_3_^−^, respectively. This oxidation peak decreased sharply in the third cycle and disappeared in the sixth cycle, which made the CV curve almost coincide with that obtained on the blank one, indicating the clearance of adsorbed I^−^. In addition, it can be seen from the figure that the generated IO_3_^−^ cannot be reduced to I^−^ again because there was no new reduction peak that appeared.

To verify the effect of the cleaning procedure, a series of control experiments were carried out on the substrate before and after the cleaning procedure. First, the interface characteristics of the electrodes before and after cleaning were verified, and Figure 2b shows the EIS spectra of the GCA electrode before and after cleaning. The EIS (Figure 2b) of the cleaned electrode had a much smaller arc (curve a) than that of the uncleaned one, suggesting a lower surface resistance to [Fe(CN)_6_]^3−/4−^ due to the removal of the impurities from the surface of the GCA electrodes. As shown in Figure 2c, CVs of 5 mmol/L potassium ferricyanide and a couple of quasi-reversible redox peaks at 0.314 and 0.218 V were detected on the cleaned GCA electrode. As for the uncleaned GCA electrode, a noticeable increase in the peak separation (0.417 V vs. 0.096 V) was observed, accompanied by a decrease in the peak current. These results could be attributed to the blocking effect of the contaminants on the electron transfer between the electrode surface and the redox couple. It also proved that the electrode cleaning procedure could effectively remove impurities on the electrode surface. Electrode cleaning was more prominent when the GCA was used as a substrate for surface-enhanced Raman scattering (SERS). In the control experiment, two electrodes before and after the cleaning procedure were immersed in 1 μmol/L methylene blue (MB) solution and then blown dry for SERS detection; the results are shown in Figure 2d. On the uncleaned GCA substrate, the SERS signal of the MB can hardly be distinguished from the fluorescence background. However, on the cleaned substrate, the background decreased from ~30,000 to ~6500 counts, and the SERS intensity of the MB molecules was significantly improved.

### 3.3. Electrochemical Characterization of the PQT@GCA Electrode

**Figure 3:**
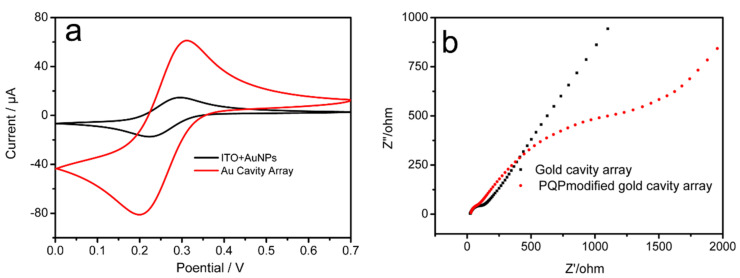
(**a**) CV curves of 5 mmol/L K_3_[Fe(CN)_6_] on a bare Au electrode and the GCA electrode, (**b**) EIS curves of GCA electrode and PQT@GCA electrode. The scan rate is 0.1 V/s.

Figure 3a shows the CV curves of K_3_[Fe(CN)_6_] on a bare Au and a GCA electrode. For the preparation of the bare Au electrode, AuNPs were first synthesized according to previous literature [41] (see Figure S2 for the TEM image of AuNPs), and then these gold nanoparticles were electrostatically adsorbed to the PDDA-modified ITO surface. Both electrodes were covered with prepunched tape to keep the electrode area consistent. As can be seen, both CV curves had one pair of well-defined redox peaks, with the peak-to-peak separation of ΔE_p_ = ~86 mV, suggesting fast electron and mass transfer on the electrode surface. Moreover, the current obtained on the GCA was about eight times larger than that on the bare Au electrode. This additional increase in current could be attributed to the specific cavity morphology and the rough surface of the gold nanoparticles that made up the GCA electrode.

The AuNPs generated by electrodeposition increase the specific surface area and provide support for the attachment of the PQT molecules. Alkanethiols containing self-assembled monolayers (SAM) have attracted extensive attention in electrochemistry. This electrode modification manner anchors the electroactive substances on the surface of the metal electrodes with covalent bonds and presents an ordered distribution [42]. EIS was used to analyze the kinetics of the electrode process and provide evidence of the successful modification of PQT molecules on the GCA. The high-frequency region is the kinetic process of the electrode reaction. The fitted semicircle diameter corresponds to the confined electron transfer process and is proportional to the resistance of the transferred electrons during the diffusion process. The Nyquist plots of the GCA electrode and the PQT@GCA electrode are shown in Figure 3b; the impedances are plotted on both real and imaginary axes as a semicircle. Curve a is composed of a very small semicircle (r_1_ = 153 Ω) and an oblique line in the low-frequency region, which is almost a straight line, indicating the resistance of the gold spherical cavity to electron transfer is very small. After the PQT molecules were modified on the GCA surface, the diameter of the semicircle formed in the high-frequency region increased significantly (r_2_ = 1200 Ω), indicating that the GCA surface was covered with PQT molecules, which hindered the direct electron transfer on the electrode surface. The result proved that the PQT@GCA electrode was successfully constructed.

### 3.4. Electrochemical Performance of the PQT@GCA Electrode

**Figure 4:**
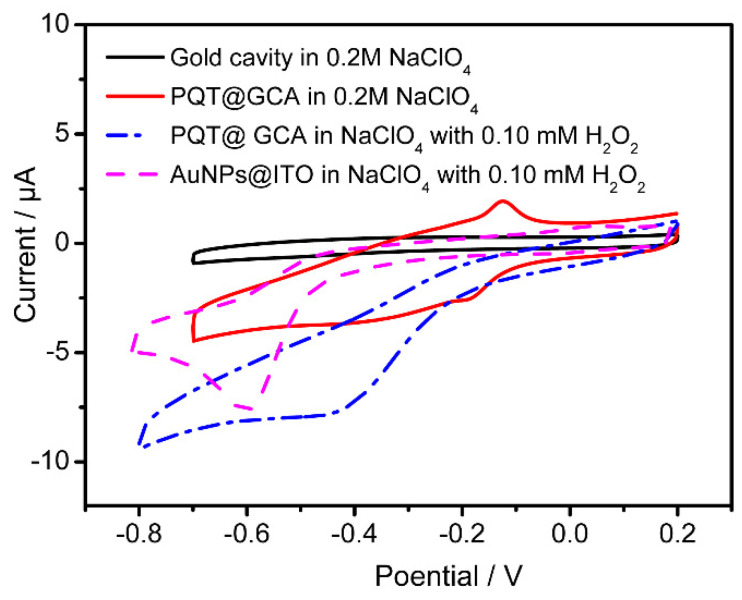
CV curves of the GCA electrode, PQT@GCA, and AuNPs@ITO electrode in 0.2 mol/L NaClO_4_ with and without H_2_O_2_.

Typical CV curves of GCA electrodes and PQT@GCA electrodes in different electrolytes were collected to verify the electrocatalytic performance of the PQT@GCA for H_2_O_2_ detection. As shown in Figure 4, no redox peak was observed at the GCA electrode in 0.2 mol/L NaClO_4_. For the PQT-modified GCA electrode, a pair of redox peaks appeared. The oxidation and reduction peaks were located at −0.13 and −0.19 V, corresponding to the direct electron transfer between the active site of the PQT molecules and the GCA electrode. The ΔE_p_ = 60 mV, and the ratio of the oxidation current to the reduction current Ipa/Ipc was 1.26 ± 0.05, implying a quasi-reversible two-electron electrochemical reaction for the fixed PQT molecules.

The sulfhydryl groups in the PQT molecules formed Au-S bonds and were firmly immobilized on the GCA surface. The coverage of the electroactive substance on the GCA electrode could be calculated according to the Laviron Equation (1) [43].

(1) $$I_{P}=\frac{nFQv}{4RT}=\frac{n^{2}F^{2}A\Gamma v}{4RT}$$

where *I_p_* is the peak current, n is the number of transferred electrons, and *F*, *R*, and *T* represent the Faraday constant, the gas constant, and the thermodynamic temperature, respectively, while *ν* stands for the scanning rate, and *Q* is the quantity of charge (*C*) calculated from the peak area of the cyclic voltammograms. In the present case, *ν* = 0.2 V/s, *I_p_* = 5.36 × 10^−6^ A, *Q* = 1.257 × 10^−6^ C, and then *n* = 2.09 can be obtained. Thus, the concentration of the electroactive substance Γ was calculated to be 7.65 × 10^−11^ mol/cm^2^.

**Scheme 1:**
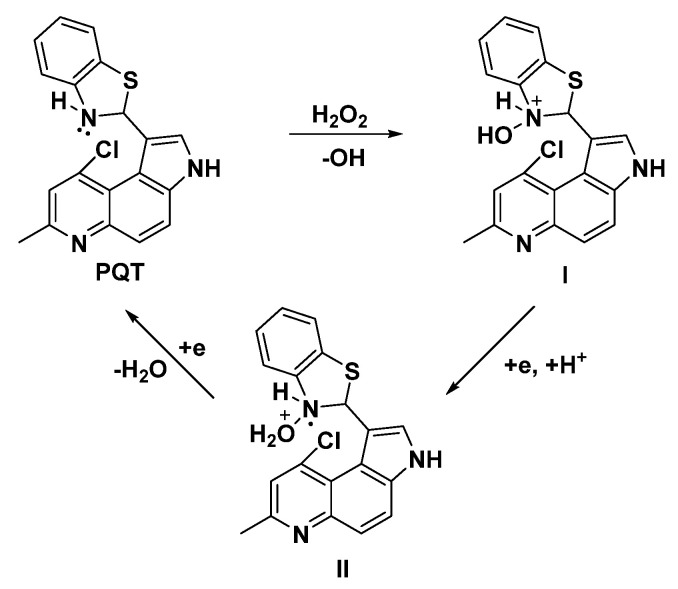
Schematic diagram of the possible mechanism of the reaction between PQT molecule and H_2_O_2_.

After adding H_2_O_2_ to the electrolyte, its reduction potential on the surface of the GCA electrode is located at about −0.65 V. In contrast, the CV curve on the PQT@GCA electrode changed significantly, manifesting as a greatly enhanced reduction current, a positive shift of the peak potential to −0.4 V, and the disappearance of the oxidation current. These phenomena were typical features of electrocatalysis occurring on the electrode surface, proving that the PQT molecules play an electrocatalytic role in reducing H_2_O_2_. We suggest the possible electrocatalytic mechanism on the PQT@GCA as Scheme 1. The compound PQT could be oxidized by hydrogen peroxide and produce the hydroxylammonium ion I; the intermediate I captures an electron and a proton to form the anion radical II, and the intermediate II can gain an electron and get rid of a water molecule to regenerate the starting compound PQT. In a word, the compound PQT is the catalyst in the electrochemical detection process.

**Figure 5:**
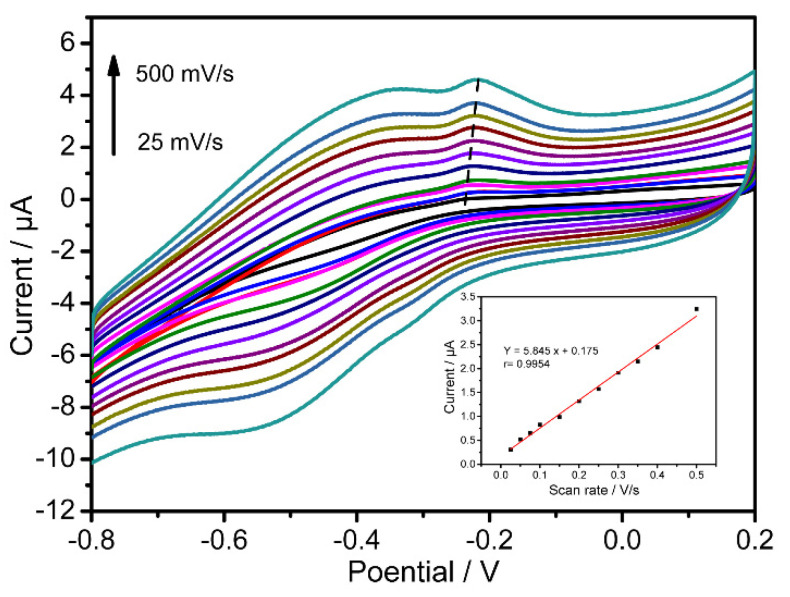
CV curves of PQT@GCA electrode at various scan rates from 25 mV/s to 0.5 V/s. The inset is the linear relationship between scan rate and oxidation peak current.

The electrochemical performance of PQT on the GCA electrode was used to study the electrode surface dynamics mechanism. Figure 5 shows the CV curves of the PQT@GCA electrode in 0.2 mol/L NaClO_4_. As observed, the oxidation and reduction peak currents of the PQT molecules become more significant with the increase of the scan rate. The inset in Figure 5 further shows the linear relationship between the peak currents and the scan rates in 25 to 500 mV s^−^^1^ with the linear regression equations y = 5.845x + 0.175 (r = 0.9954). The results indicated that the electron transfer between the PQT molecules and the GCA surface was a surface-controlled process. Additionally, there is only a slight increase in the ΔE_p_, suggesting a fast electron transfer rate for the immobilized PQT.

**Figure 6:**
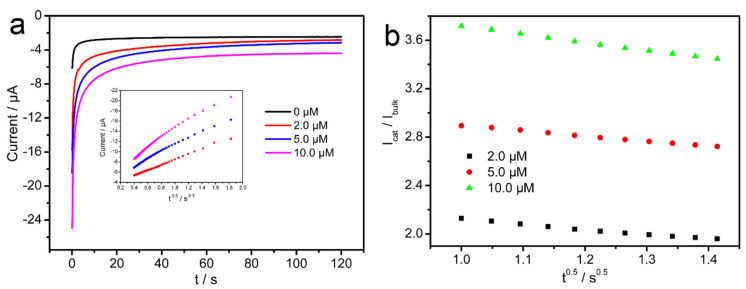
(**a**) Current–time curves on the PQT@GCA electrode in 0.2 mol/L NaClO_4_ containing different glucose concentrations. The inset is the linear relationship between *t*^−0.5^ and the current. (**b**) The linear relationship between the square roots of time and the catalytic current (*I_cat_*) ratio to blank current (*I_bulk_*).

Next, we investigated the performance of detecting glucose by chronoamperometry. To perform these detections, different concentrations of glucose (0, 2.0, 5.0, and 10.0 μmol/L) were reacted with glucose oxidase in advance. The PQT@GCA electrode was then introduced to the electrolyte, the potential was set as 0.2 V, and the time–current curves were collected. As shown in Figure 6a, the steady-state current is achieved within 10 s, indicating a shorter response time as a glucose sensor. The diffusion coefficient (*D*) for H_2_O_2_ can be obtained according to the Cottrell Equation (2) [44].

(2) $$I_{t}=\frac{nFAD^{1/2}C_{0}}{\pi^{0.5}t^{0.5}}$$

where *I_t_* is the catalytic current of the PQT@GCA electrode in the presence of glucose (*A*), *A* is the area of the GCA electrode exposed to the electrolyte (cm^2^), and *C*_0_ represents the concentration of glucose (mol/mL). There is an excellent linear relationship between the reciprocal of the square root of time (*t*^−0.5^) and the current at different concentrations (inset of Figure 6a). The slope of the straight line is equal to *nFACπ*^−0.5^*D*^0.5^, and it can be calculated that the average diffusion coefficient *D* of H_2_O_2_ on the surface of the PQT@GCA electrode is 2.73 × 10^−6^ cm^2^·s^−1^.

The catalytic rate constant *K_cat_* of PQT can be estimated by plotting the dependence of *I_cat_/I_buff_* to t^1/2^ at different concentrations according to the following Equation (3) [45]:

(3) $$\frac{I_{cat}}{I_{L}}=[\gamma^{0.5}\pi^{0.5}erf(\gamma^{0.5})+exp(\frac{-\gamma }{\gamma })]$$

where *I_cat_* and *I_l_* are the currents of the GCA@PQT electrode in the presence or absence of glucose (H_2_O_2_), *γ* = *kC_0_t* is the parameter of the error function, *k* is the catalytic rate constant, *C*_0_ is the concentration of glucose (H_2_O_2_), and *t* is the running time when *γ* is gradient to 1, so we get the following Equation (4).

(4) $$\frac{I_{cat}}{I_{L}}=\pi^{0.5}{\left(kC_{0}t\right)}^{0.5}$$

As plotted in Figure 6b, the average catalytic rate constant was calculated as 1.72 × 10^4^ M^−1^ s^−1^.

### 3.5. Electrochemical Determination of Glucose

Glucose oxidase (GOX) can catalyze the oxidation of glucose to gluconic acid, and oxygen as the oxidant will be reduced to H_2_O_2_ after reaction:

C_6_H_12_O_6_ (GLU) + O_2_ + H_2_O + GOX = 2C_6_H_12_O_7_ + H_2_O_2_ (5)

We thus used the response of the PQT molecules to the generated H_2_O_2_ to achieve the indirect detection of glucose content in various samples. SWV was used for quantitative analysis because it suppressed the background current and had a high signal-to-noise ratio.

**Figure 7:**
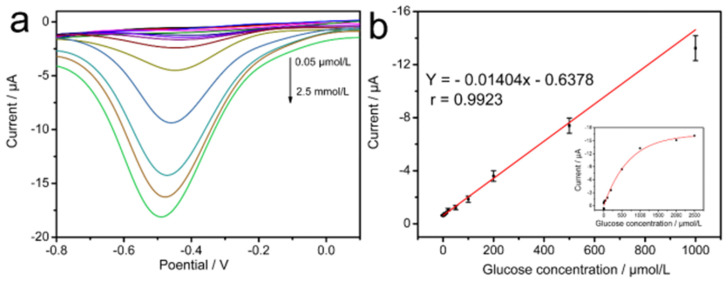
(**a**) SWVs of PQT@GCCA electrode in 0.2 mol/L NaClO_4_ at different glucose concentration (0.05 µmol/L–2.5 mmol/L). (**b**) The relationship between glucose concentration and reduction current; inset shows the linear relationship between glucose concentration and reduction current between 0.05 µmol/L–1.0 mmol/L.

As shown in Figure 7a, the reduction current gradually increased with the increased glucose concentration. There is a good linear relationship between the current value and the H_2_O_2_ concentration, ranging from 0.2 μmol/L–1.0 mmol/L (Figure 7b). The linear equation is I(µA) = −0.0145C (µmol/L)—0.6378 (r = 0.97). The limit of detection was calculated to be 0.05 μmol/L (S/N = 3), using IUPAC definition LOD = 3sb/q, where sb is the standard deviation of the blank signal and q is the slope of the calibration curve.

**Figure 8:**
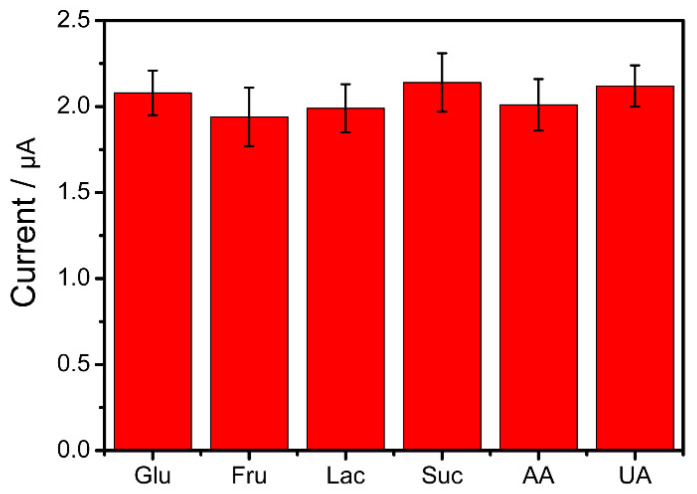
Specificity of the glucose sensor examined by addition of several interferents to GLU solution. Each error bar represents the standard deviation with *n* = 4.

We parallelly prepared four GCAs using the same electrodeposition parameters and took 50 μmol/L glucose standard solution as the detection object, while the relative standard deviation (RSD) of the interassay result was used to assess the reproducibility of the GCA electrode preparation. The RSD of five consecutive determinations on each electrode was used to evaluate the repeatability of the data. The results showed that the PQT-based glucose sensor had satisfactory reproducibility (RSD = 8.3%) and data repeatability (RSD = 11.3%). To investigate the selectivity of this sensor, we introduced high concentrations (2 mmol/L) of sucrose, lactose, fructose, uric acid, and ascorbic acid into a 100.0 μmol/L glucose solution. As shown in Figure 8, the changes in the current signal before and after introducing the interfering components are negligible, indicating the excellent selectivity of the proposed sensor in glucose detection.

### 3.6. Preliminary Analysis of Actual Samples

Finally, to demonstrate the applicability of the PQT@GCA sensor in actual samples, we measured the glucose levels in the serum samples of the five volunteers and compared them with the test results in the hospital. To ensure the concentration of the spiked sample within the linear range of the method, we spiked a 0.1 mL serum sample with 20.0 and 50.0 μL of the 10 mmol/L glucose solution, and then diluted it to 10.0 mL. The comparison of the glucose concentration detection results in the serum samples between the clinical method and the as-proposed method, as well as the recovery and RSD results, are listed in Table 1. The results obtained from the PQT@GCA electrode were consistent with those from the clinical approach. The recovery of this method was 97.7–105.8%, and the RSD was 7.3–10.2%, implying the high accuracy of the proposed PQT@GCA-based method in actual samples for glucose detection.

## 4. Conclusions

In conclusion, we presented a novel PQT@GCA electrode for the electrochemical determination of H_2_O_2_/glucose. The GCA electrode was fabricated by a multicurrent pulse technology, and the electrodeposited AuNPs gradually filled the crevices of the close-packed PS spheres template to form the final high-ordered cavity array structure. The PQT is a kind of pyrroloquinoline skeleton containing organic molecules. The oxidation of benzothiazoline to benzothiazole makes the PQT@GCA have an electrocatalytic effect on the reduction of H_2_O_2_, thereby realizing the electrochemical detection of reactive oxygen-related species. In the range from 0.2 μmol/L to 1.0 mmol/L, the glucose concentration had an excellent linear relationship with the current value, and the minimum detection limit of the method could reach 0.05 μmol/L. Finally, the proposed glucose sensor works well in human serum samples, proving its high clinical diagnosis accuracy.

## Figures and Tables

**Table 1:** Recovery of glucose in spiked serum samples.

No.	Clinical Methods (mmol/L)	This Method (mmol/L)	Added (mmol/L)	Found (mmol/L) (ng/mL) (*n* = 4)	RSD (%)	Recovery (%)
1 *	4.6	4.31	2.0	6.23	10.2	98.7
2	6.2	6.48	2.0	8.69	8.7	102.5
3	5.5	5.27	5.0	10.84	9.4	105.8
4	11.3	11.43	5.0	16.65	7.3	101.3
5	13.6	14.52	5.0	19.07	7.8	97.7

* The reference value of the clinical method is 3.0–6.0 mmol/L.

## Data Availability

Data are contained within the article or Supplementary material.

## Supplementary Materials

The following supporting information can be downloaded at: https://www.mdpi.com/article/10.3390/nano12101770/s1, Figure S1: Synthetic route of PQT; Figure S2: TEM image of gold nanoparticles; Figure S3: ^1^H NMR spectra of compound 2; Figure S4: ^1^H NMR spectra of compound PQT; Figure S5: ^13^C NMR spectra of compound PQT; Figure S6: HRMS spectra of compound PQT. References [46,47,48,49] are cited in the Supplementary Materials
