# Supplementary material for: H2O2/Glucose Sensor Based on a Pyrroloquinoline Skeleton-Containing Molecule Modified Gold Cavity Array Electrode
Source: Nanomaterials (Basel). 2022 May 23;12(10):1770. doi: 10.3390/nano12101770 (PMC9144773; doi:10.3390/nano12101770)
Supplement: Supplementary file 1 [file nanomaterials-12-01770-s001.zip › nanomaterials-1727500-supplementary.pdf]

Supplementary Materials

H_2O_2/Glucose Sensor Based on a Pyrroloquinoline Skeleton-Containing Molecule Modified Gold Cavity Array Electrode

Kaiyue Wang, Xuefang Gu *, Qun Zhao, Xinyi Shao, Yaqi Xiao, Chongyu Zhong, Shu Tian * and Bing Yang *

School of Chemistry and Chemical Engineering, Nantong University, Nantong 226019, China; 1608022036@stmail.ntu.edu.cn (K.W.); qunzhao@ntu.edu.cn (Q.Z.); 1908110265@stmail.ntu.edu.cn (X.S.); 1908110186@stmail.ntu.edu.cn (Y.X.); 1908110353@stmail.ntu.edu.cn (C.Z.)

* Correspondence: xuefang818@ntu.edu.cn (X.G.); tian0429@ntu.edu.cn (S.T.); yangbing111@ntu.edu.cn (B.Y.)

S1. Synthetic procedures and characterization of compounds

S1.1. Materials and apparatus

All commercially available chemicals were used without further purification. Chromatographic purification of products was performed on silica gel (200-300 mesh). Melting point was determined on a micro melting point apparatus (SGW X-4B, Shanghai, China) and uncorrected. ^1H and ^{13}C NMR spectra were measured with a Bruker AVANCE III HD 400M spectrometer with Tetramethyl silane (TMS) as the internal standard. Chemical shifts (δ) were shown in ppm (parts per million) with respect to TMS. Coupling constants (J) were reported in Hz. HRMS (High Resolution Mass Spectrometry) data were obtained from an AB Sciex TripleTOF 4600 System mass spectrometer with an ESI (electrospray ionization) source.

SEM images were taken on a Zeiss Gemini sem300 field emission scanning electron microscope. Electrodeposition and electrochemical measurements were recorded at a CHI 660E electrochemical station (Shanghai, China). Platinum and Ag | AgCl (3M KCl) electrodes were used as auxiliary and reference electrodes; before the H_2O_2 detection, deoxygenate with argon for at least 15 minutes. In the case of standard glucose solutions and actual samples, deoxygenation was performed after reacting with GOX.

SERS spectra were measured on a Jobin Yvon/HORIBA HR320 Raman spectrometer with an integral BX 41 confocal microscope (Olympus). The excitation source was a 532 nm laser with a D_2 attenuation filter. The laser power at the sample was ~0.1 mW, and the accumulation time was 5 s.

S1.2. Fabrication of highly-ordered gold cavity arrays (GCA)

ITO glass slides were cut to 1×3 cm and then cleaned by ultrasonic in water, ethanol, isopropanol and chloroform for 15 minutes, respectively. A 10 nm-thick Cr layer and a 100 nm-thick Au layer were electromagnetically sputtered on the conductive surface of the cleaned ITO slide.

For the assembly of a monolayer of PS spheres, a water/ethanol (volume ratio 1:1.5) dispersion containing 1.5 μm-diameter monodisperse PS spheres (5 wt %) was thoroughly dispersed by ultrasonic, and injected by a microperistaltic pump onto the surface of water through a hydrophilic silicon wafer (in a 15-cm-diameter petri dish). To reduce the surface tension of water and to prevent the PS spheres from spreading in a disordered manner, ~100 μL of 2 wt. % sodium dodecyl sulfate aqueous solution was dropped onto the water surface in advance. The suspension flowed down along the silicon wafer slowly and evenly, forming a

close-packed film on the water surface. The petri dish was placed in a non-interference environment for 24 h, so that the PS particles suspended in water could be sedimentated. A piece of gold-coated ITO slide was obliquely inserted below the liquid level at an angle of 30°, and then carefully drawn out of the water slowly. The spheres were transferred onto the surface of the glass slide with the arrangement of PS well maintained. The glass slide with PS template on its surface was then dried at 90 °C for 1 h to remove the remaining solvent.

The PS template-coated substrate was then used as the working electrode for further electrodeposition of gold in a plating bath solution containing 0.029 mol L^{-1} $HAuCl_4$ solution with 1.267 mol L^{-1} sodium sulfite (Na_2SO_3), 0.173 mol L^{-1} potassium hydrogen phosphate (K_2HPO_4) and 0.017 mol L^{-1} EDTA according to the literature [1,2]. The gold arrays were produced using multi-current pulse plating with the first pulse to a current density of 30 mA cm^{-2} for 100 ms, followed by a train of pulses of 5 mA cm^{-2} for 60 ms separated by a rest time of 1 s (zero current). Then, the electrode was immersed into toluene to dissolve the PS spheres, leaving an ordered array of interconnected sphere segment voids.

S1.3. Chemical syntheses

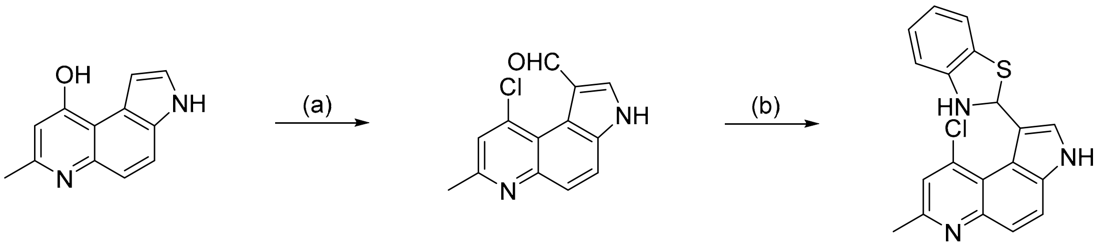

Figure S1. Synthetic route of PQT. Reagent and condition: (a) $POCl_3$, DMF, 30 ℃, 8 h, 90%; (b) 2-aminobenzenethiol, MeOH, reflux, 1.5 h, 80%.

S1.3.1. Synthesis of 3*H*-9-chloro-7-methyl-1-formyl-pyrrolo[3,2-*f*]quinoline (**2**).

According to previous reports [3,4], 3*H*-9-Hydroxy-pyrrolo[3,2-*f*]quinoline (**1**) was synthesized. Compound **1** (0.2032 g, 1.0 mmol) was dissolved in DMF (1.5 mL), and phosphorus oxychloride (1.0 mL) was then added dropwise in an ice bath. The reaction mixture was stirred at 0℃for 15 min and stirred at 30℃ for 8 h. After the completion of the reaction was confirmed by TLC, the reaction was quenched by ice water (7.5 mL) in an ice bath and the solution was adjusted to pH = 8-9 with ammonia water (5.0 mL). A brown precipitate was observed and the precipitate was filtered by diatomite media. Finally, the crude product was purified on silica gel column to afford compound **2** as a yellow solid (yield: 90%). m.p. 159.0-161.0 °C. ^{1}H NMR (400 MHz, DMSO-d_6) δ 2.64 (s, 3H), 7.64 (s, 1H), 7.78 (d, 1H, J = 8.8 Hz), 7.93 (d, 1H, J = 9.2 Hz), 8.17 (d, 1H, J = 2.8 Hz), 10.48 (s, 1H), 12.73 (s, 1H). These ^{1}H NMR data were in agreement with literature [5].

S1.3.2. Synthesis of 2-(9-chloro-7-methyl-3*H*-pyrrolo[3,2-*f*]quinolin-1-yl)-2,3-dihydrobenzo[*d*]thiazole (**PQT**).

To a methanol solution (15.0 mL) of compound **2** (0.2445g, 1.0 mmol) was added 2-aminobenzenethiol (0.15 mL), and the mixture was stirred and heated to reflux for 1.5 h. After confirming the consumption of compound **2** by TLC analysis, the reaction mixture was evaporated under reduced pressure. The residue was scratched, washed with EtOAc (10 mL) under stirring and filtered. The precipitate was washed with EtOAc/EtOH (v/v, 1/1) and dried to afford the pure product **PQT** as a yellow solid (yield: 80%). m.p. 156.0-158.0 °C. ^{1}H NMR (400 MHz, CD_3OD) δ 2.79 (s, 3H), 7.35-7.46 (m, 3H), 7.55-7.63 (m, 4H), 7.92 (d, 1H, J = 7.2 Hz), 8.02 (d, 1H, J = 9.2 Hz). ^{13}C NMR (100 MHz, CD_3OD) δ 21.1, 71.0, 101.4, 106.2, 109.4, 113.2, 119.2, 121.4, 121.8, 124.4, 125.9, 126.9, 129.3, 130.3, 133.2, 133.6, 140.0, 150.6, 155.4. HRMS (ESI-TOF) *m*/*z*: [M-Cl]$^+$ Calcd. for $C_{19}H_{14}N_3S$ 316.0903, Found 316.0911.

S2. TEM image

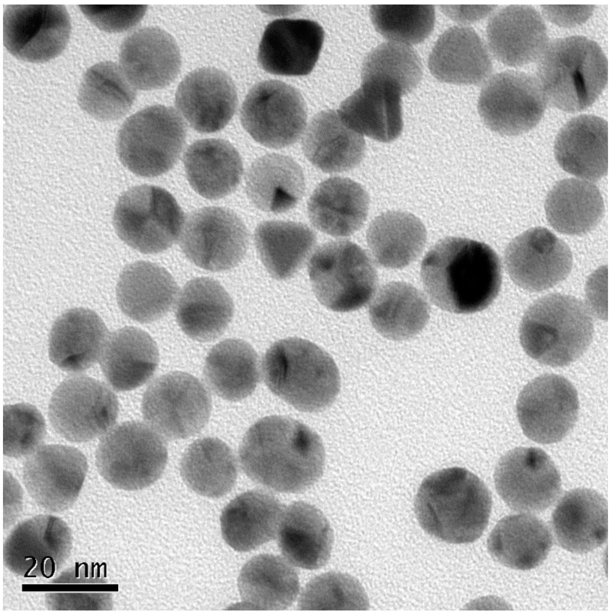

Figure S2. TEM image of gold nanoparticles.

S3. NMR and HRMS spectra of compounds

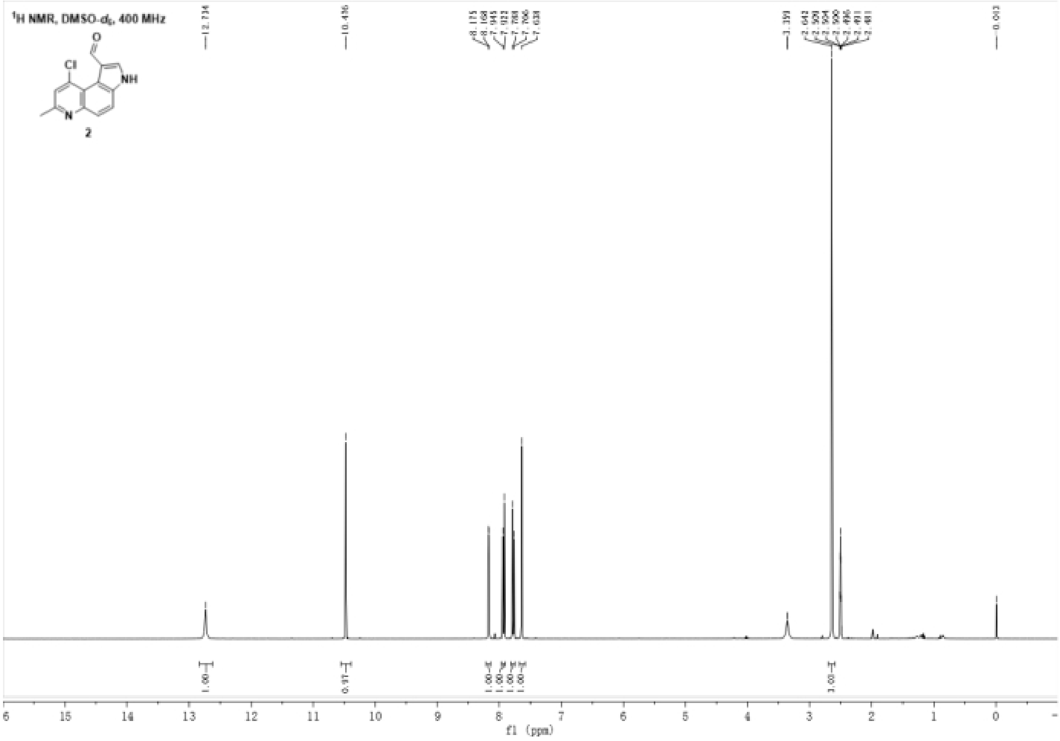

Figure S3. ^{1}H NMR spectra of compound 2.

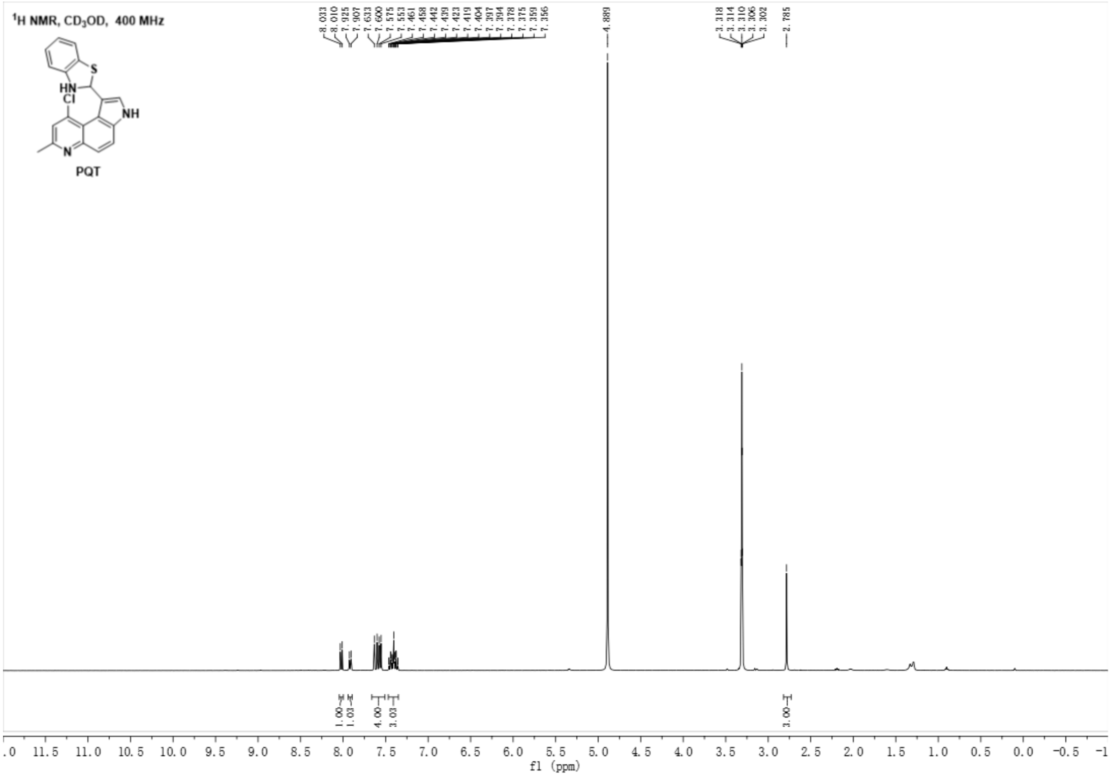

Figure S4. ^{1}H NMR spectra of compound PQT.

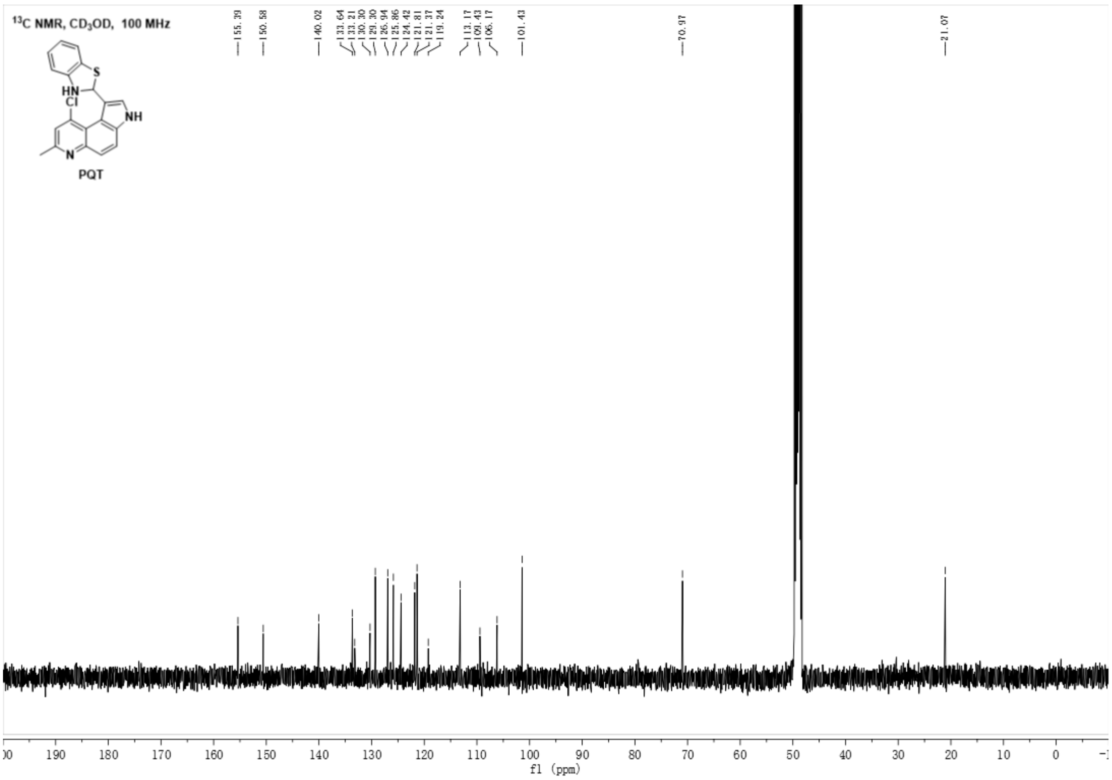

Figure S5. ^{13}C NMR spectra of compound PQT.

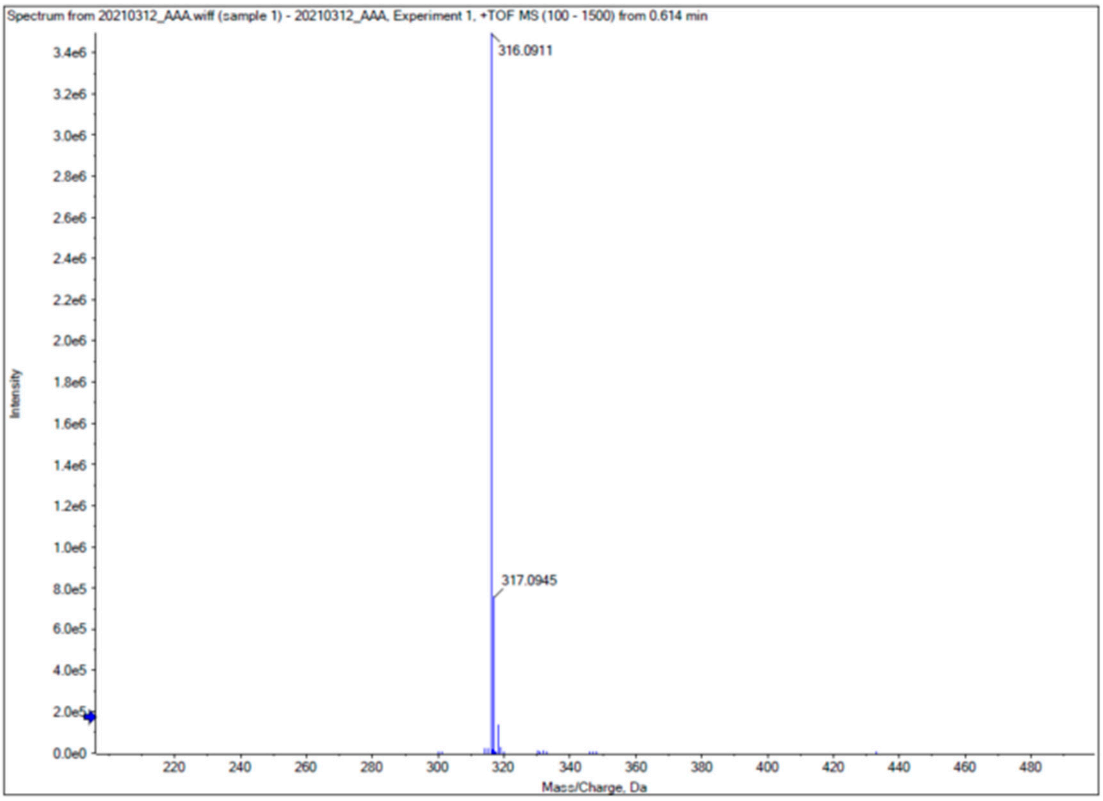

Figure S6. HRMS spectra of compound PQT.

References

1. Bartlett, P.N.; Baumberg, J.J.; Coyle, S.; Abdelsalam, M.E. Optical properties of nanostructured metal films. *Faraday Discuss.* **2004**, *125*, 117-132.
2. Fan, D.; Wu, S.; Tian, S.; Zhou, J.; Ju, Y.; Ma, C.; Shi, J. Detection of dopamine on a poly(metanilic acid) decorated two-dimensional gold cavity array electrode. *RSC Adv.* **2014**, *4*, 49560-49568.
3. Ferlin, M.; Gatto, B.; Chiarelotto, G.; Palumbo, M. Pyrrolo-quinoline derivatives as potential antineoplastic drugs. *Bioorgan. Med. Chem.* **2000**, *8*, 1415-1422.
4. Ferlin, M.; Gatto, B.; Chiarelotto, G.; Palumbo, M. Novel pyrrolo [3, 2-f] quinolines: synthesis and antiproliferative activity. *Bioorgan. Med. Chem.* **2001**, *9*, 1843-1848.
5. Yang, B.; Zhou, J.; Huang, X.; Chen, Z.; Tian, S.; Shi, Y. A new pyrroloquinoloo line-derivative-based fluorescent probe for the selective detection and cell imaging of lysine. *Pharmaceuticicals* **2022**, *15*, 474.
